# Normal-tension glaucoma and obstructive sleep apnea syndrome: a prospective study

**DOI:** 10.1186/1471-2415-14-27

**Published:** 2014-03-10

**Authors:** Gorkem Bilgin

**Affiliations:** 1 Ophthalmologist, Hacettepe University Gün Hospital, Beytepe, 06800 Ankara, Turkey

**Keywords:** Normal-tension glaucoma, Obstructive sleep apnea

## Abstract

**Background:**

Today, identified risk factors for normal-tension glaucoma (NTG) include abnormal ocular blood flow, abnormal blood coagulation, systemic hypotension, ischemic vascular disorders, and autoimmune diseases. However, pathogenesis of the condition remains unclear. On the other hand, there are also a few studies suggesting that the obstructive sleep apnea syndrome (OSAS) may compromise optic nerve head perfusion and cause glaucomatous optic neuropathy by creating transient hypoxemia and increasing vascular resistance. In this study, we evaluated the possible association between OSAS and NTG.

**Methods:**

We recruited 24 patients with NTG and 24 age and sex matched controls who were also similar for systemic risk factors such as diabetes mellitus (DM), hypertension (HT) and hypercholesterolemia. All patients and controls underwent over-night polysomnography (PSG) for the diagnosis of OSAS and calculation of Apnea-Hypopnea Index (AHI).

**Results:**

Patients and controls were statistically similar in terms of age, sex, gender, smoking, systemic risk factors, neck circumference and body mass index. The subjects with AHI ≥ 20 were accepted as OSAS. Ten (41.7%) of 24 patients with NTG and 3 (12.5%) of 24 controls had OSAS (p < 0.05).

**Conclusions:**

The prevalence of OSAS was higher in patients with NTG and the difference between patient and control groups was statistically significant (p < 0.05).

## Background

Normal-tension glaucoma (NTG) is an optic neuropathy associated with a glaucomatous optic nerve head, progressive retinal nerve fiber layer thinning, characteristic visual field defects, open anterior chamber angle on gonioscopy, and a maximum intraocular pressure (IOP) below 21 mmHg [1]. Currently, identified risk factors for NTG include abnormal ocular blood flow, abnormal blood coagulation, systemic hypotension, ischemic vascular disorders, and autoimmune disease [2-4]. However, pathogenesis of the condition remains unclear.

Obstructive sleep apnea syndrome (OSAS) is a common yet underdiagnosed condition that may be associated with significant morbidity if left untreated. It is characterized by recurrent interruption of normal breathing during sleep, owing to upper airway obstruction (apneic spells) [5]. The apneic spells can cause a decrease in the arterial oxygen saturation and a rise in the carbon dioxide saturation during sleep. OSAS has been associated with many vascular diseases including cardiovascular disease, hypertension and stroke [6-8].

It is also suggested that OSAS could create transient hypoxemia and increase vascular resistance, which may compromise optic nerve head perfusion and oxygenation and cause glaucomatous optic neuropathy [3,9]. In this study, we evaluated the possible association between OSAS and NTG.

## Methods

This prospective study involved 24 patients with NTG (age range 53-78 years; 9 males and 15 females) who were recruited from general ophthalmology clinics. Twenty-four age and sex matched subjects without any kind of glaucoma diseases (age range 53-77 years; 9 males and 15 females) who were also similar for systemic risk factors such as diabetes mellitus (DM), hypertension (HT) or hypercholesterolaemia were selected from internal medicine clinics and taken as control group. This study followed the tenets of the Declaration of Helsinki and informed consent was obtained from all subjects. The study was approved by local ethics committee.

The diagnosis of NTG was made based on the following criteria:

1- A cup-to-disc ratio (c/d) over 0.5 or difference of c/d between two eyes >0.2 with thinning of the neuroretinal rim.

2- Glaucomatous visual field defects such as localized defects, paracentral scotoma, Bjerrum scotoma, nasal step, temporal sector defect, and diffuse defect which cannot be explained by any neurologic or fundus lesion.

3- Open iridocorneal angle.

4- IOP < 21 mmHg without treatment.

The patients were required to fulfill all 4 criteria to make the diagnosis of NTG.

All the patients in NTG group had at least 5 visual field tests and the participants in the control group had two visual field tests before the recruitment to the study. Both NTG patients and the controls and all the visual fields were reviewed by two glaucoma specialists and one neuro-ophthalmologist who were masked to the diagnosis of the subjects.

For NTG patients; mean IOP was 16.2 ± 2.4 mmHg and mean c/d ratio was 0.548 ± 0.135 . The Mean deviation value and pattern standard deviation value of the patients’ visual fields were found to be -5.1 ± 3.2 and 5.6 ± 2.7 decibels, respectively. Three (12.5%) of 24 NTG patients had disc haemorrhages during the diagnosis. On the other hand, mean IOP was 11.6 ± 1.8 mmHg and mean c/d ratio was 0.365 ± 0.092 for the controls. There were no field defects in the visual field tests of controls.

Participants underwent overnight PSG recordings in two sleep laboratories. Sleep was continuously recorded on a computerized system (Grass Technologies, West Warwick, Rhode Island, USA) scored in 30-sec epochs according to American Academy of Sleep Medicine (AASM) standardized criteria [10]. Apnea during sleep was defined as cessation of airflow (90% fall in the amplitude of airflow signal compared to the baseline airflow) lasting at least 10 seconds. Hypopnea was defined as a 50% or greater fall in airflow lasting ten or more seconds associated with a 3% or greater fall in oxygen saturation from baseline. The apnea–hypopnea index (AHI) was calculated using the total number of respiratory events (apneas and hypopneas) per hour sleep [11]. Subjects with an AHI ≥20 were regarded as having OSAS.

Means were compared by Student’s *t* test or Mann-Whitney *U* test. Prevalence of OSAS in patients with NTG was compared with the matched controls by using Fisher exact test. Comparison of the clinical and polysomnographic characteristics of the patients with NTG and matched controls was performed by using the unpaired *t* test or Fisher exact test. SSPS version 17.0 for Windows (SSPS Inc., Chicago, Illinois, USA) was used for statistical analysis. Significance was accepted for p < 0.05.

## Results

Mean age of the patients with NTG and matched controls were 66.7 ± 10.2 and 67.2 ± 11.6 years, respectively. There were no statistically significant differences with regard to age, gender, systemic risk factors, smoking, neck circumference and body-mass index (BMI) between the two groups (p > 0.05). This data is summarized in Tables 1, 2.

**Table 1:** Clinical characteristics of patients with NTG and control group

	**Patients with NTG (n = 24)**	**Controls (n = 24)**
**Age (years)**	66.7 ± 10.2	67.2 ± 11.6
**Male**	9 (37.5%)	9 (37.5%)
**Female**	15 (62.5%)	15 (62.5%)
**Smoking**	13 (54.2%)	11 (45.8%)
**Body-mass index (kg/m**^**2**^**)**	26.8 ± 2.3	25.9 ± 2.4
**Neck circumference (cm)**	34.61 ± 2.43	33.26 ± 3.78

NTG: Normal-tension glaucoma.

**Table 2:** Systemic diseases of patient and control groups

	**Patients with NTG (n = 24)**	**Controls (n = 24)**
**Hypertension**	14 (58.3%)	13 (54.2%)
**Diabetes mellitus**	6 (25%)	4 (16.7%)
**Hypercholesterolaemia**	14 (58.3%)	12 (50%)
**Coronary artery disease**	4 (16.7%)	4 (16.7%)

NTG: Normal-tension glaucoma.

Ten (41.7%) of 24 patients with NTG and 3 (12.5%) of 24 subjects in the control group met criteria for OSAS (AHI ≥ 20); the difference was statistically significant (p < 0.05, Fisher exact test). Relative risk for sleep apnea in NTG patients was 3.34 compared to subjects in the control group.

All of the 10 patients who were diagnosed with OSAS in our study started treatment in sleep disorder clinics.

## Discussion

Although the pathogenesis of NTG remains uncertain, the bulk of evidence points to a chronic progressive ischemic disorder with hypoperfusion of the optic nerve head as a mechanism of the disorder (3). On the other hand, intermittent upper airway obstructions in OSAS during sleep would cause hypoxia with subsequent decrease in PaO_2_ and increase in PaCO_2_. The episodic vascular insufficiency may compromise optic nerve perfusion and oxygenation and subsequently cause optic neuropathy [12]. Sergi et al. [13] reported that the decrease in RNFL thickness correlated with the severity of OSAS. Kargi et al. [14] measured the RNFL with a scanning laser polarimeter and found that retinal nerve fiber layer (RNLF) thickness was reduced in patients with OSAS. They suggested that decreased ocular perfusion related to hypoxia and vasospasm associated with OSAS may cause RNFL thinning.

A further support for the relationship between NTG and OSAS was reported by Kremmer et al. who demonstrated a positive influence of OSAS therapy with nasal continuous positive airways pressure (nCPAP) ventilation on both disease courses [15].

The presence of these possible links between OSAS and NTG has led to investigations into the prevalence of OSAS in NTG patients. Mojon et al. [16] observed OSAS in 7 of the 16 NTG patients (44%). However, their controls were all male subjects from a previously published study. Thus, they did not use an age and sex matched control group. Marcus et al. [17] described the rate of sleep apnea among patients with NTG as 55.5%. They did not observe any OSAS cases in their control group. In another prospective study of 6 patients with known NTG and symptomatic snoring, Blumen Ohana et al. [18] reported that 3 of the patients subsequently diagnosed with OSAS on PSG testing. However, both Marcus et al. and Blumen Ohana et al. offered PSG only to those who reported a positive history of sleep disturbance or snoring. Since subjective reports often underestimate the prevalence of OSAS, this may create a statistical bias in both of these studies.

In our study, we compared our OSAS prevalence in NTG patients with age and sex matched controls. To the best of our knowledge, this is the first study with age and sex matched control cases for evaluating OSAS prevalence in NTG patients. Both of our groups were also similar regarding BMI, neck circumference, smoking and systemic risk factors that can lead to OSAS (Tables 1, 2). In addition to these, we believe that by using a cutoff point of 20 for AHI, we were able to reduce false-positive findings and provide a more accurate assessment of association between OSAS and NTG. We found an increased prevalence of OSAS in patients with NTG. Relative risk for OSAS in NTG patients was 3.34 compared to subjects in the control group.

In NTG, treatment is indicated for patients who have visual field loss and rapid progression. Management is directed toward the implementation of a lower IOP or to the correction of reversible circulatory deficiencies at the optic nerve with the treatment of vasospasm, nocturnal hypotension and carotid insufficiency. We think that OSAS also compromises circulation at the optic nerve head by causing hypoxia and vascular dysregulation in patients with NTG and, the glaucomatous damage was shown to be stable after nCPAP treatment [15]. Thus, OSAS may be a treatable cause of circulatory deficiency in the optic nerve head.

Our small sample size was the limitation of this study. We offered overnight PSG test to both our NTG patients and to the control subjects. During the study, we realized that people in our region did not have much information and knowledge about overnight PSG. Thus, it was very difficult to convince all the participants about the necessity of the PSG test. In light of our relatively small sample size, further prospective studies using PSG test in larger number of subjects are required to validate the association between NTG and OSAS.

## Conclusions

In conclusion, OSAS should be considered as a significant risk factor for NTG. We think that it is advisable to take an accurate sleep history (including questions about snoring, nocturnal gasping-choking, daytime sleepiness and morning headaches) from patients with NTG and refer these patients for PSG test and nCPAP therapy.

## Abbreviations

OSAS: Obstructive sleep apnea syndrome; NTG: Normal-tension glaucoma; DM: Diabetes mellitus; HT: Hypertension; PSG: Polysomnography; AHI: Apnea-Hypopnea Index; IOP: Intraocular pressure; nCPAP: Nasal continuous positive airways pressure.

## Competing interests

As authors, we declare that we have no competing interests.

## Authors’ contributions

GB participated in the design of the study, performed the statistical analysis, helped to draft the manuscript and conceived of the study. All authors read and approved the final manuscript.

## Pre-publication history

The pre-publication history for this paper can be accessed here:

http://www.biomedcentral.com/1471-2415/14/27/prepub
